# Tungstic acid-functionalized Fe_3_O_4_@TiO_2_: preparation, characterization and its application for the synthesis of pyrano[2,3-*c*]pyrazole derivatives as a reusable magnetic nanocatalyst†

**DOI:** 10.1039/c8ra06886k

**Published:** 2018-12-06

**Authors:** Jamileh Etemad Gholtash, Mahnaz Farahi

**Affiliations:** Department of Chemistry, Yasouj University Yasouj Iran 75918-74831 farahimb@yu.ac.ir (+98) 7412242167

## Abstract

A new magnetic nanocatalyst based on the immobilization of tungstic acid onto 3-chloropropyl-grafted TiO_2_-coated Fe_3_O_4_ nanoparticles (Fe_3_O_4_@TiO_2_@(CH_2_)_3_OWO_3_H) was prepared, characterized, and applied for the synthesis of pyrano[2,3-*c*]pyrazole derivatives. The characterization was performed using FT-IR spectroscopy, X-ray diffraction (XRD), scanning electron microscopy (SEM), energy-dispersive X-ray spectroscopy (EDS), and vibrating sample magnetometry (VSM) analysis. Pyranopyrazoles were synthesized in the presence of this novel catalyst *via* a three-component reaction of 3-methyl-1-phenyl-2-pyrazolin-5-one, malononitrile, and aromatic aldehydes with high yields. It is a low cost, nontoxic and thermally stable catalyst, which shows a long life and can be reused for several catalytic cycles without deactivation or selectivity loss.

## Abstract 2

A new nanocatalyst based on the immobilization of tungstic acid onto 3-chloropropyl-grafted TiO_2_-coated Fe_3_O_4_ nanoparticles (Fe_3_O_4_@TiO_2_@(CH_2_)_3_OWO_3_H) was prepared, characterized, and applied for the synthesis of pyrano[2,3-c]pyrazoles.

## Graphical abstract

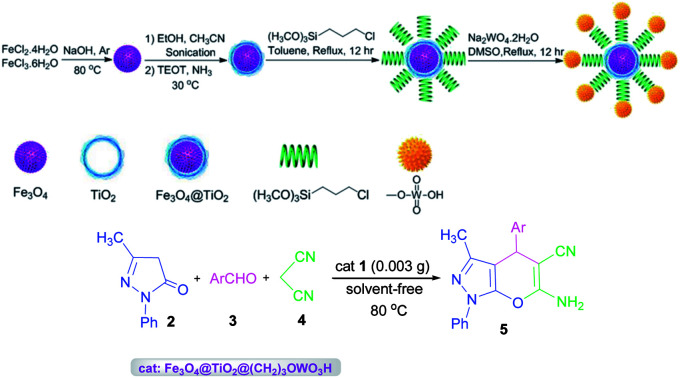

## Introduction

Despite the many benefits of homogeneous catalysts such as high activity and selectivity, heterogeneous catalytic systems have surpassed them due to the high capability for recycling and reutilization.^1^ Over the past century, nanocatalysts as a bridge between heterogeneous and homogeneous catalysis have drawn great attention for application in different fields. The significant advantage of nanoparticles is their high specific surface area to volume ratio leading to increase in the contact with the reactants.^2–4^ They also have many other advantages such as presence of various surface reactive sites, high activity, selectivity, and desired resilience, which make them recognized as a pioneering technology in green chemistry.^5^ Despite the several mentioned benefits of nanomaterials, they are difficult to separate. Therefore, it is important to design recoverable and well-dispersed nanocatalysts.

Recently, Fe_3_O_4_ magnetite nanoparticles (MNPs) have been intensively used as catalytic supports owing to the facility of isolation from the reaction mixture using an external magnet. Furthermore, these systems possess highly potential active sites for loading of other functional groups to prepare novel heterogeneous catalysts.^6–11^ To prevent Fe_3_O_4_ nanoparticles from oxidation in an air atmosphere and in order to increase the surface area and simplify the surface functionalization, a protective shell can be formed on their surface.^12^

TiO_2_ have been successfully applied as a nontoxic, low cost and highly effective catalyst that has good mechanical resistance and stability in acidic and oxidative environments. TiO_2_ was also found to be a good support material for heterogeneous catalysis due to the strong metal support interaction, chemical stability, and acid–base property.^13–15^ Because of the small size of TiO_2_ particles and difficulties in the separation of catalyst from the reaction media, there are some significant drawbacks in using TiO_2_ as a heterogeneous catalyst. Immobilization of TiO_2_ on magnetic nanoparticles as suitable alternative supports to produce magnetically recoverable heterogeneous catalysts allows a convenient recovery of magnetic catalyst under an external magnetic field.^16,17^

In recent years, there has been increasing attention in the synthesis of pyrano[2,3-*c*]pyrazoles as an important class of heterocyclic compounds.^18–21^ This interest has resulted from their vital biological activities such as analgesic, antitumor, anticancer, and anti-inflammatory as well as their potential as inhibitors of human Chk1 kinase.^22,23^ The pyrano[2,3-*c*]pyrazoles subunit is present in various pharmaceutical and medicinally useful molecules.^24^ They are also the main building blocks in the synthesis of natural products as well as in heterocyclic compounds.^25^

Taking the above facts into consideration and in continuation of our research on the synthesis of heterogeneous catalysts,^26–31^ in this study, we have immobilized tungstic acid on TiO_2_-coated Fe_3_O_4_ magnetic nanoparticles (Fe_3_O_4_@TiO_2_@(CH_2_)_3_OWO_3_H) and then investigated its performance as a novel strong, recoverable, and stable acid nanocatalyst for synthesis of pyrano[2,3-*c*]pyrazole derivatives.

## Experimental

Chemicals were purchased from Fluka, Merck and Aldrich chemical companies. Known products were identified *via* comparison of their structural data and physical properties with their reported data in the literature. Melting points were determined by an electrothermal kSB1N apparatus. Fourier transform infrared (FT-IR) spectra were recorded on a Shimadzu-470 spectrometer using KBr pellets. The morphology of the particles was observed by scanning electron microscopy (SEM) under acceleration voltage of 26 kV. X-ray powder diffraction (XRD) patterns were recorded using a Bruker AXS (D8 Advance) X-ray diffractometer with Cu Kα radiation (*λ* = 0.15418 nm). Energy dispersive spectroscopy (EDS) was obtained using TESCAN Vega model instrument. The magnetic measurement was carried out in a vibrating sample magnetometer (VSM; Kashan university, Kashan, Iran) at room-temperature.

### Preparation of Fe_3_O_4_ MNPs

A solution of FeCl_2_·4H_2_O (1 g, 5 mmol) and FeCl_3_·6H_2_O (2.7 g, 10 mmol) (dissolved in 45 mL double distilled water) was degassed with an argon gas, heated to 80 °C and stirred for 30 minutes. Then, sodium hydroxide solution (5 mL, 10 M) was slowly added dropwise. After stirring at 80 °C for 1 h under argon atmosphere, Fe_3_O_4_ MNPs was separated by an external magnet and washed with double distilled water until pH 9 and then dried at 60 °C.^32^

### Synthesis of nano-Fe_3_O_4_@TiO_2_

The prepared Fe_3_O_4_ MNPs were dispersed in a mixture of absolute ethanol and acetonitrile (125 : 45 mL) by sonication for 20 min. Then, ammonia aqueous solution (0.75 mL, 25%) was added under vigorous stirring for 30 minutes. After that, tetraethyl orthotitanate (TEOT) (1.5 mL) dissolved in absolute ethanol (20 mL) was slowly added to the above suspension, under continuous mechanical stirring at 30 °C. This mixture was stirred for 1.5 h to obtain nano-Fe_3_O_4_@TiO_2_. The resulting precipitate was collected by an external magnet and washed with absolute ethanol and dried at room-temperature.^33^

### Preparation of Fe_3_O_4_@TiO_2_@(CH_2_)_3_Cl

A mixture of Fe_3_O_4_@TiO_2_ (0.2 g) and 3-chloropropyltrimethoxysilane (2 mL) was stirred with an argon gas under reflux conditions for 12 h. Next, the obtained product was separated by a normal magnet and washed using toluene, ethanol–water mixture, and distilled water. Finally, obtained Fe_3_O_4_@TiO_2_@(CH_2_)_3_Cl was dried in an oven at 60 °C.

### Preparation of Fe_3_O_4_@TiO_2_@(CH_2_)_3_OWO_3_H (1)

In the final stage, a mixture of Fe_3_O_4_@TiO_2_@(CH_2_)_3_Cl (0.5 g) and Na_2_WO_4_·2H_2_O (0.25 g) in DMSO (5 mL) was stirred at reflux under argon atmosphere for 12 h. After cooling, the obtained catalyst was decanted and washed twice with DMSO, once with distilled water, and dried at 60 °C for 6 h. Then, the functionalized magnetic nanoparticles were added to the flask containing HCl (30 mL, 0.1 N) and stirred for 1 h at room-temperature. The resulting catalyst was decanted, washed with DMSO and water, and finally dried in oven at 60 °C for 12 h.

### General procedure for the synthesis of pyranopyrazole derivatives 5

Fe_3_O_4_@TiO_2_@(CH_2_)_3_OWO_3_H (0.003 g) was added to a mixture of aldehydes (1 mmol), 3-methyl-1-phenyl-2-pyrazolin-5-one (1 mmol), and malononitrile (1 mmol). The mixture was stirred under solvent-free conditions at 80 °C for the requisite time. The reaction progress was screened using TLC. After completion of the reaction, ethanol (5 mL) was added to the reaction mass and the catalyst was collected with an external magnet. Additional purification was achieved by recrystallization from hot ethanol.

#### 6-Amino-4-(2,4-dihydroxyphenyl)-3-methyl-1-phenyl-1,4-dihydropyrano[2,3-*c*]pyrazole-5-carbonitrile (5q)

White crystals, IR (KBr): *ν*_max_ = 3424, 3309, 3183, 2923, 2854, 2211, 1590, 1511, 1400, 1351, 1255, 1189, 1045, 842, 800 cm^−1^. ^1^H NMR (400 MHz, DMSO-*d*_6_): *δ* = 9.68 (s, 1H), 7.92 (s, 1H), 7.76 (s, 2H), 7.52–7.75 (m, 2H), 7.25–7.27 (m, 2H), 6.89 (s, 2H), 6.45 (d, 2H, *J* = 4 Hz), 6.32 (s, 1H), 5.53 (s, 1H), 1.82 (s, 3H). ^13^C NMR (100 MHz, DMSO-*d*_6_): *δ* = 166.61, 162.25, 161.98, 159.71, 155.85, 153.63, 138.27, 126.38, 123.14, 117.11, 114.33, 108.82, 107.09, 103.83, 101.35, 75.76, 42.11, 26.12. Anal. calcd for C_20_H_16_N_4_O_3_: C, 66.66; H, 4.48; N, 15.55. Found: C, 66.58; H, 4.53; N, 15.60.

#### 6-Amino-4-(5-bromo-2-hydroxyphenyl)-3-methyl-1-phenyl-1,4-dihydropyrano[2,3-*c*]pyrazole-5-carbonitrile (5r)

Yellow crystals, IR (KBr): *ν*_max_ = 3455, 3347, 3212, 2935, 2210, 1646, 1608, 1554, 1477, 1388, 1280, 1222, 1087, 802, 470 cm^−1^. ^1^H NMR (400 MHz, DMSO-*d*_6_): *δ* = 8.71 (s, 1H), 7.71 (d, 2H, *J* = 4 Hz), 7.52 (t, 2H, *J* = 4 Hz), 7.36 (s, 1H), 7.27–7.30 (m, 2H), 6.83–6.93 (m, 2H), 6.61 (d, 1H, *J* = 4 Hz), 5.46 (s, 1H), 2.04 (s, 3H). ^13^C NMR (100 MHz, DMSO-*d*_6_): *δ* = 162.97, 162.31, 158.22, 158.17, 151.67, 140.98, 137.22, 127.62, 121.35, 118.38, 118.06, 118.03, 117.24, 116.73, 97.66, 75.22, 47.56, 23.87. Anal. calcd for C_20_H_15_BrN_4_O_2_: C, 56.75; H, 3.57; N, 13.24. Found: C, 56.70; H, 3.51; N, 13.29.

## Results and discussion

**Scheme 1:**
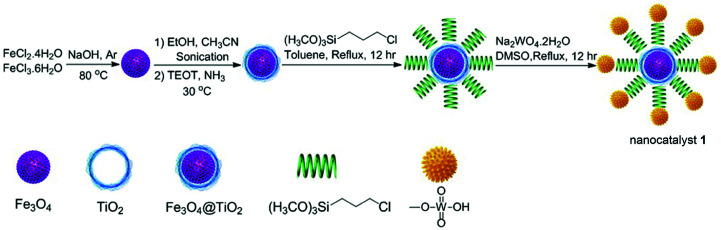
Preparation of Fe_3_O_4_@TiO_2_@(CH_2_)_3_OWO_3_H (1).

Fe_3_O_4_@TiO_2_@(CH_2_)_3_OWO_3_H (1) was prepared following the protocol shown in Scheme 1. Firstly, Fe_3_O_4_ nanoparticles were synthesized *via* coprecipitation method.^32^ Subsequently, tetraethyl orthotitanate (TEOT), as a coating agent, reacts with Fe_3_O_4_ to develop TiO_2_ layer on the Fe_3_O_4_ backbone.^33^ The OH groups on the titanium coating magnetic nanoparticles (Fe_3_O_4_@TiO_2_) can be functionalized with 3-chloropropyltriethoxysilan molecule. Finally, the chloride group was replaced by tungstic acid to prepare Fe_3_O_4_@TiO_2_@(CH_2_)_3_OWO_3_H (1) as a magnetic nanocatalyst. The obtained catalyst was characterized *via* FT-IR, X-ray diffraction patterns (XRD), scanning electron microscopy (SEM), energy-dispersive X-ray spectroscopy (EDS), and vibrating sample magnetometer (VSM) analysis.

**Fig. 1:**
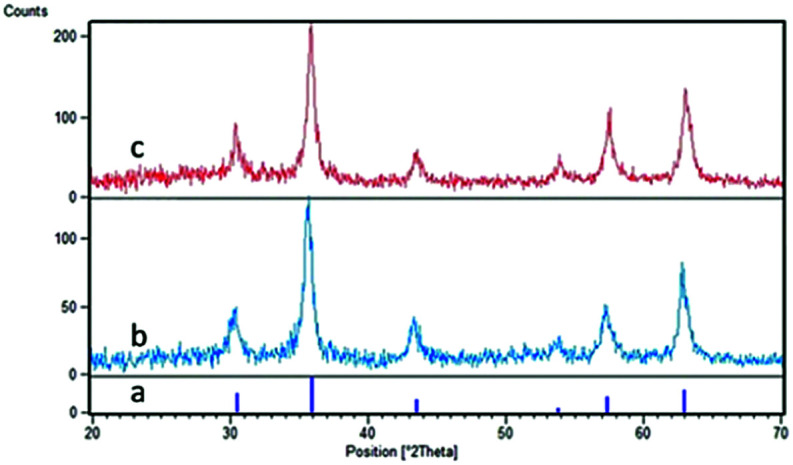
The XRD patterns of (a) Fe_3_O_4_, (b) Fe_3_O_4_@TiO_2_, and (c) Fe_3_O_4_@TiO_2_@(CH_2_)_3_OWO_3_H.

Fig. 1 shows the X-ray diffraction (XRD) patterns of the synthesized nano-Fe_3_O_4_@TiO_2_ and Fe_3_O_4_@TiO_2_@(CH_2_)_3_OWO_3_H in the range 20–70°. In Fig. 1c, the following signals at (220), (210), (400), (511), and (440) and 2*θ* = 30.10°, 35.60°, 43.40°, 53.50°, 57.20°, and 62.80 planes confirm that the main formed phase was a cubic Fe_3_O_4_, which is in agreement with the JCPD 79-0417 standard.^34^ Also, the mentioned indexes revealed that the new catalyst in Fig. 1c has the similar structure to Fe_3_O_4_ nanoparticles in Fig. 1a, and this shows that no phase change was observed after surface modification of the magnetite nanoparticles. The peak that confirmed the presence of tungstate group appeared in the range of 2*θ* = 22°.^35^ The particle size of the prepared catalyst could be approximated using the Debye–Scherrer equation, *D* = *kλ*/*β* cos *θ*, where *D* is the average crystalline size, *λ* is the X-ray wavelength, *k* is the Scherrer constant, *β* is the half width of XRD diffraction lines, and *θ* is the Bragg diffraction angle. The particle size relevant to the Debye–Scherrer equation was calculated to be 23 nm.

**Fig. 2:**
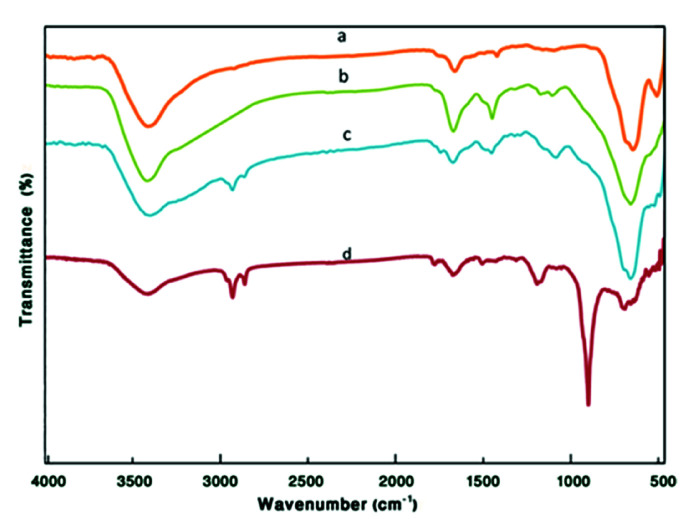
The FT-IR spectra of (a) Fe_3_O_4_ MNPs, (b) Fe_3_O_4_@TiO_2_, (c) Fe_3_O_4_@TiO_2_@(CH_2_)_3_Cl, and (d) Fe_3_O_4_@TiO_2_@(CH_2_)_3_OWO_3_H.

**Fig. 3:**
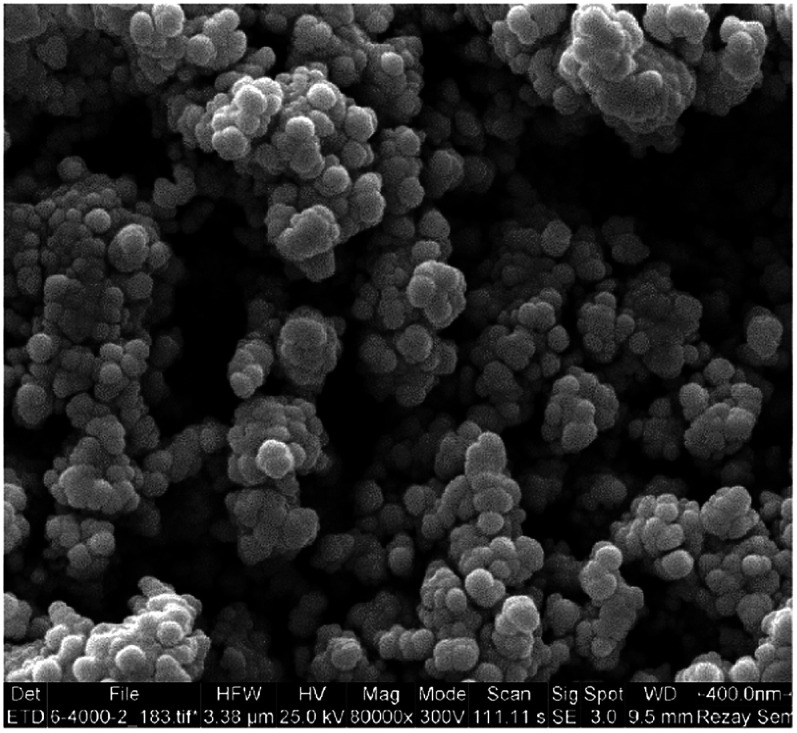
The SEM image of catalyst 1.

The FT-IR spectra of the Fe_3_O_4_, Fe_3_O_4_@TiO_2_, Fe_3_O_4_@TiO_2_@(CH_2_)_3_Cl, and Fe_3_O_4_@TiO_2_@(CH_2_)_3_OWO_3_H are shown in Fig. 2. The broad peak at about 2600–3700 cm^−1^ could be attributed to the overlapping of OH stretching bands (corresponding to uncoated OH and acidic OH). The presence of characteristic peaks corresponding to Fe–O stretching vibration near 584 cm^−1^ in all compared spectra was a confirmation of how nanostructure of Fe_3_O_4_ was preserved throughout the process. In Fig. 2b, the peaks discerned at 1118 cm^−1^ and 1400 cm^−1^ can be ascribed to the stretching vibration modes of Ti–O and Fe–O–Ti bonds, respectively. In Fig. 2c, CH_2_ bending, as a broad band and symmetric CH_2_ and asymmetric CH_2_ of the alkyl chains appeared at 1480 cm^−1^ and 2860–2923 cm^−1^, respectively. In Fig. 2d, after modification of Fe_3_O_4_@TiO_2_@(CH_2_)_3_Cl with tungstic acid, new bonds appeared at 887 cm^−1^, which corresponded to W=O vibrations (Fig. 3).

**Fig. 4:**
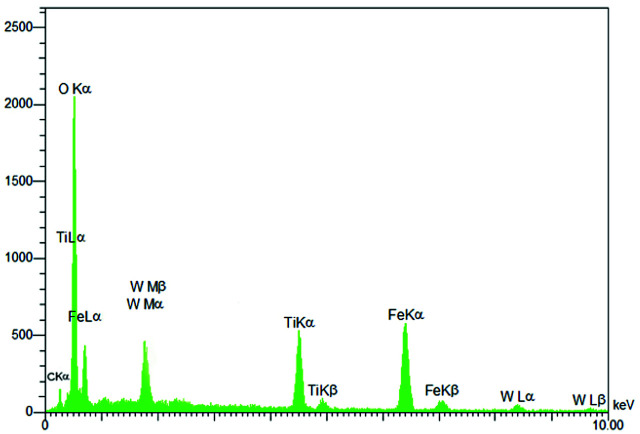
EDS analysis of Fe_3_O_4_@TiO_2_@(CH_2_)_3_OWO_3_H.

Surface morphology of Fe_3_O_4_@TiO_2_@(CH_2_)_3_OWO_3_H was observed *via* scanning electron microscopy (SEM). The result demonstrates that the sample consists of homogeneous spherical nanoparticles with diameters in the range of 33.79–90.72 nm (Fig. 4).

Energy-dispersive X-ray spectroscopy (EDS) was used for the structural characterization of Fe_3_O_4_@TiO_2_@(CH_2_)_3_OWO_3_H. As can be seen in Fig. 4, the components of this catalyst included Fe, Ti, O, C and W, which indicate the acceptable concordance with the expectations and also confirm the successful incorporation of tungstate groups.

**Fig. 5:**
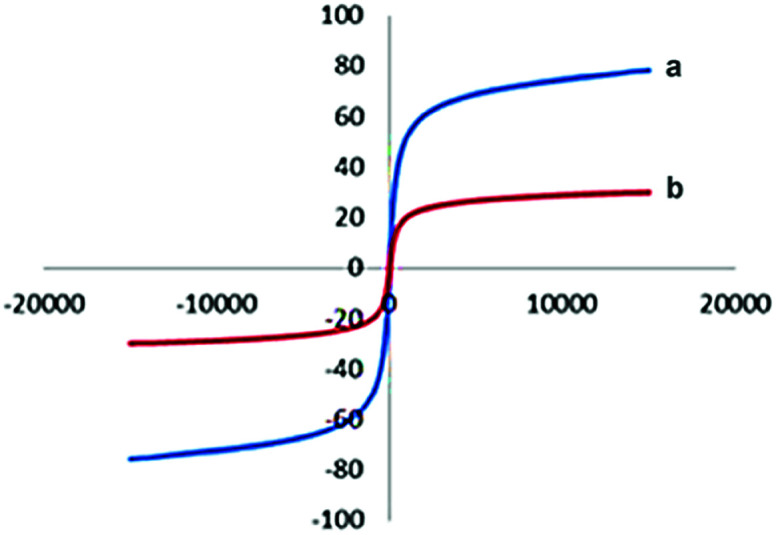
Room-temperature magnetization curves of (a) Fe_3_O_4_@TiO_2_ and (b) Fe_3_O_4_@TiO_2_@(CH_2_)_3_OWO_3_H.

The vibrating sample magnetometer (VSM) was applied to evaluate the magnetic measurement of the prepared catalyst (Fig. 5). In the present method, the magnetic behavior of the above catalyst has been calculated by drawing the hysteresis loops at room-temperature. The below magnetization curves shows the saturation magnetization of Fe_3_O_4_@TiO_2_ nanoparticles and Fe_3_O_4_@TiO_2_@(CH_2_)_3_OWO_3_H, which were diminished to 23.4 emu g^−1^ from 65.8 emu g^−1^ for Fe_3_O_4_@TiO_2_. As can be seen, the difference of saturation magnetization between Fe_3_O_4_@TiO_2_ nanoparticles and Fe_3_O_4_@TiO_2_@(CH_2_)_3_OWO_3_H was small.

**Scheme 2:**
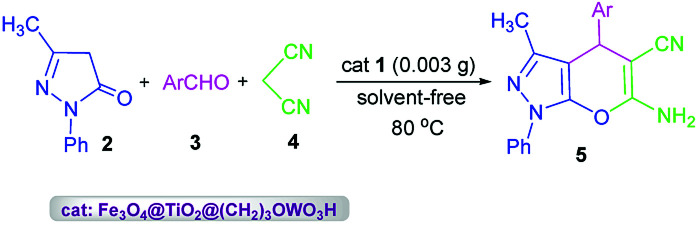
Synthesis of pyranopyrazoles 5 in the presence of nanocatalyst 1.

After full structural characterization of nano-Fe_3_O_4_@TiO_2_@(CH_2_)_3_OWO_3_H, it has been successfully applied to synthesis of pyrano[2,3-*c*]pyrazole derivatives 5*via* a three-component reaction of 3-methyl-1-phenyl-2-pyrazolin-5-one 2, aromatic aldehydes 3, malononitrile 4 under solvent-free conditions (Scheme 2).

In order to find the most appropriate reaction conditions, the reaction of 3-methyl-1-phenyl-2-pyrazolin-5-one (1 mmol), benzaldehyde (1 mmol), and malononitrile (1 mmol) was selected as a model reaction. The desired product was not produced in the absence of a catalyst even after a long reaction time. Then, we attempted with different amounts of Fe_3_O_4_@TiO_2_@(CH_2_)_3_OWO_3_H as catalyst under various conditions. The obtained data for several catalyst loads, temperatures, and solvents are summarized in Table 1. It was seen that the selection of 0.003 g of catalyst 1 at 80 °C under solvent-free conditions would be the best of choice (Table 1, entry 11).

**Table 1:** Optimization of the model reaction

Entry	Solvent	Catalyst (g)	*T* (°C)	Time (h)	Yield^a^ (%)
1	EtOH	0.005	Reflux	180	52
2	CHCl_3_	0.005	Reflux	180	50
3	Toluene	0.005	Reflux	180	45
4	H_2_O	0.005	Reflux	180	40
5	CH_3_CN	0.005	Reflux	180	40
6	MeOH	0.005	Reflux	180	50
7	—	0.005	60	180	70
8	—	0.005	80	120	75
9	—	0.005	100	120	75
10	—	0.004	80	120	80
11	—	0.003	80	75	90
12	—	0.003	100	75	85
13	—	0.003	110	75	80
14	—	0.007	80	75	85

a Isolated yields.

Under these conditions, a range of aryl aldehydes, containing both electron-donating and electron-withdrawing groups were examined and resulted in good to excellent yields of the products in short reaction times (Table 2).

**Table 2:** Fe_3_O_4_@TiO_2_@(CH_2_)_3_OWO_3_H-catalyzed synthesis of pyrano[2,3-*c*]pyrazoles 5^a^

Entry	Ar	Time (min)	Yield^b^ (%)	Mp (°C)
5a	C_6_H_5_	75	90	170–172 (ref. 36)
5b	4-OCH_3_C_6_H_4_	90	87	174–175 (ref. 36)
5c	2,4-Cl_2_C_6_H_3_	80	84	185–186 (ref. 36)
5d	4-ClC_6_H_4_	70	90	172–174 (ref. 36)
5e	2-BrC_6_H_4_	82	78	166–167 (ref. 17)
5f	3-ClC_6_H_4_	85	86	157–159 (ref. 17)
5g	2-ClC_6_H_4_	80	89	144–146 (ref. 36)
5h	4-NO_2_C_6_H_4_	70	79	194–196 (ref. 36)
5k	4-C_3_H_7_C_6_H_4_	90	88	169–170 (ref. 36)
5l	4-BrC_6_H_4_	77	78	183–184 (ref. 36)
5m	4-OHC_6_H_4_	95	92	211–212 (ref. 36)
5n	4-(PhCH_2_O) C_6_H_4_	100	80	160–161 (ref. 36)
5p	3-OC_2_H_5_ 4-OH C_6_H_3_	95	83	169–171 (ref. 36)
5q	2,4-(OH)_2_ C_6_H_3_	110	80	320–321^c^
5r	5-Br 2-OH C_6_H_3_	120	83	314–315^c^

a Reaction conditions: 3-methyl-1-phenyl-2-pyrazolin-5-one (1 mmol), arylaldehyde (1 mmol), malononitrile (1 mmol), and Fe_3_O_4_@TiO_2_@(CH_2_)_3_OWO_3_H (0.003 g), 80 °C.

b Isolated yields.

c Novel product.

**Fig. 6:**
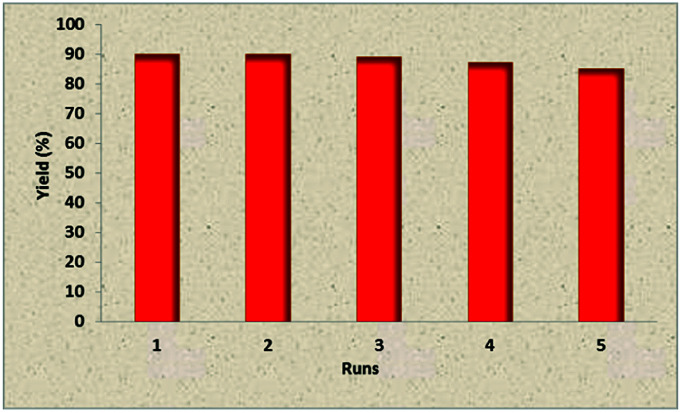
Reusability of catalyst 1 in the reaction between 3-methyl-1-phenyl-2-pyrazolin-5-one, malononitrile, and benzaldehyde.

Due to the importance of recoverability and recyclability of the catalyst from both practical and economical viewpoints, we next examined the reusability of Fe_3_O_4_@TiO_2_@(CH_2_)_3_OWO_3_H in the reaction of 3-methyl-1-phenyl-2-pyrazolin-5-one, benzaldehyde, and malononitrile under optimized reaction conditions. Fe_3_O_4_@TiO_2_@(CH_2_)_3_OWO_3_H was separated by a permanent magnet from the reaction mixture, washed with methanol, and reused in the next run without further treatment. The results revealed that the catalyst could be reused for five cycles without remarkable loss in catalytic performance. The data are exhibited in Fig. 6.

**Scheme 3:**
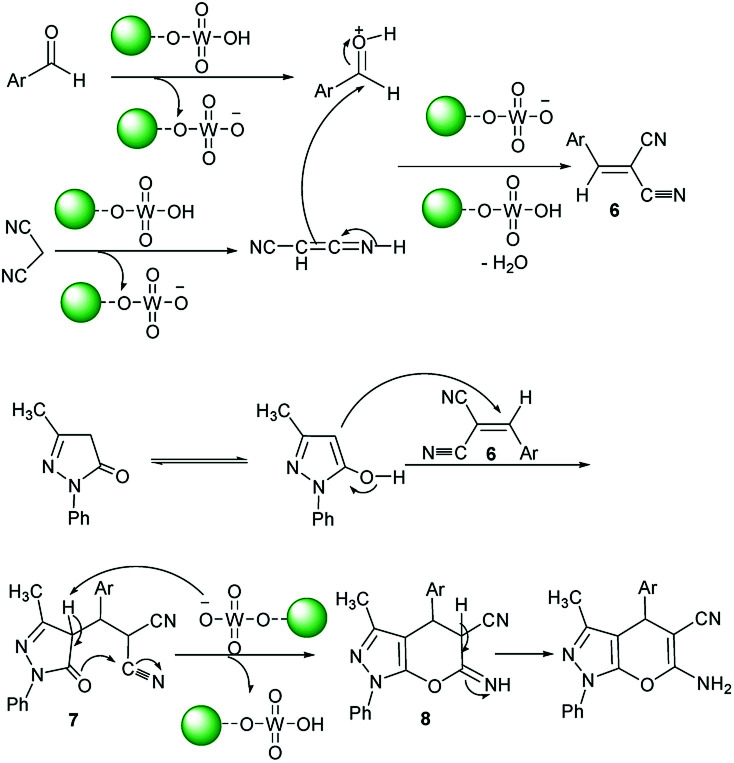
Proposed mechanism for the synthesis of product 5 catalyzed by catalyst 1.

The proposed formation mechanism of product 5 is given in Scheme 3. Initially, intermediate 6 was formed *via* the condensation of activated aromatic aldehydes and malononitrile in the presence of acid catalyst 1. It is reasonable to suppose that the intermediate 6 was attached by *c*-4 of 3-methyl-1-phenyl-2-pyrazolin-5-one which rearranged into intermediate 7. Cyclization of 7 by the nucleophilic attack of C=O group on the cyano moiety gives 8. Finally, a sequence of tautomerization of 8 generates the related product.

The major advantages of the presented protocol over existing methods can be seen by comparing our results with those of some recently reported procedures, as shown in Table 3.

**Table 3:** Comparison of the results for the synthesis of 5a by other catalysts

Entry	Catalyst	Catalyst loading	Condition	Time (min)/yield^a^ (%)
1	Piperidine	0.05 mL	EtOH, MW	8/61 (ref. 37)
2	γ-Alumina	30 mol%	H_2_O, reflux	50/80 (ref. 38)
3	Mg/Al HT	0.1 g	EtOH, r.t.	60/87 (ref. 39)
4	*p*-Toluene sulfonic acid	0.05 g	H_2_O, reflux	55/86 (ref. 40)
5	Triethylbenzylammonium chloride	0.15 g	H_2_O, 90 °C	360/99 (ref. 41)
6	Catalyst 1	0.003 g	80 °C, solvent-free	75/90^b^

a Isolated yields.

b This study.

## Conclusions

In this study, we have introduced for the first time a green and recyclable nanomagnetic solid acid catalyst, namely Fe_3_O_4_@TiO_2_@(CH_2_)_3_OWO_3_H. Structural verification was performed using FT-IR, XRD, SEM, EDS and VSM. This novel Fe_3_O_4_-based heterogeneous nanocatalyst was successfully used for the synthesis of pyrano[2,3-*c*]pyrazoles. Besides recyclability, its advantages like operational simplicity, good chemical yields combined with step- and atom-economic aspects are the main promising points of this study which make this strategy highly attractive.

## Conflicts of interest

There are no conflicts to declare.

## Supplementary Material

RA-008-C8RA06886K-s001
