# Supplementary material for: Tungstic acid-functionalized Fe3O4@TiO2: preparation, characterization and its application for the synthesis of pyrano[2,3-c]pyrazole derivatives as a reusable magnetic nanocatalyst
Source: RSC Adv. 2018 Dec 6;8(71):40962–7. doi: 10.1039/c8ra06886k (PMC9091629; doi:10.1039/c8ra06886k)

Electronic Supplementary Material (ESI) for RSC Advances.
This journal is © The Royal Society of Chemistry 2018

Supporting Information

Tungstic acid-functionalized Fe_3O_4@TiO_2: Preparation, characterization and its application for the synthesis of pyrano[2,3-*c*]pyrazole derivatives as a reusable magnetic nanocatalyst

Jamileh Etemad Gholtash and Mahnaz Farahi*

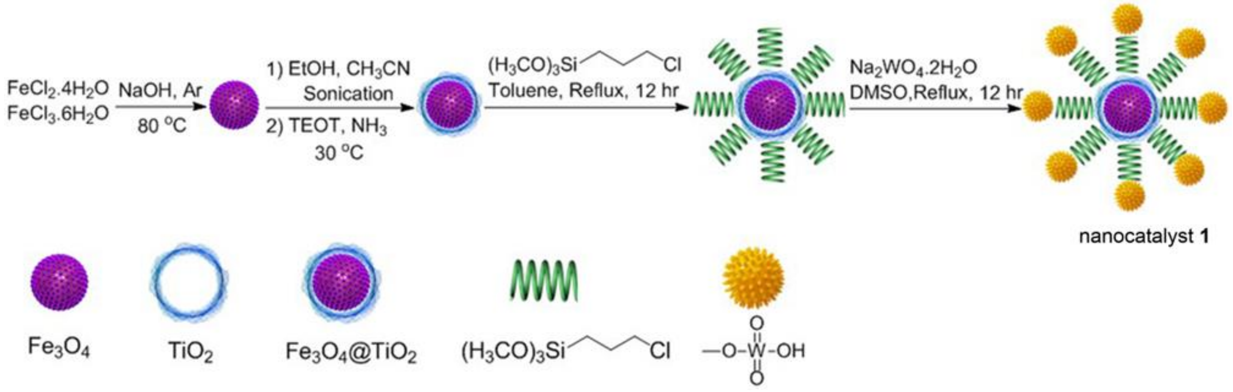

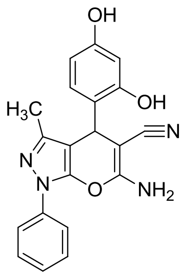
OH
OH
H_3C
N
N
O
CN
NH_2

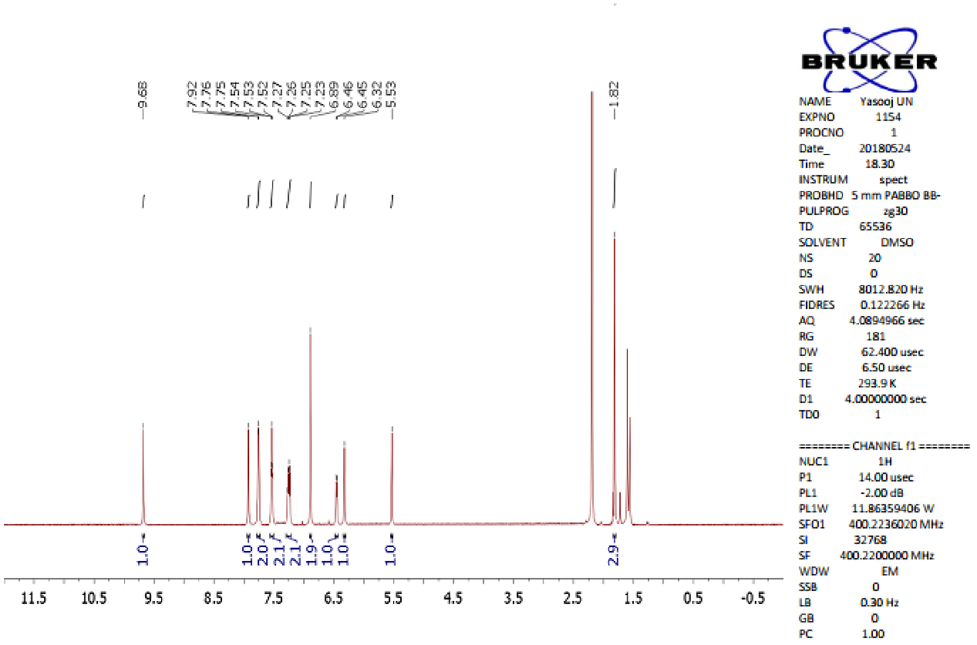
9.68
7.92
7.76
7.75
7.54
7.53
7.52
7.27
7.26
7.25
7.23
6.89
6.46
6.45
6.32
5.53
1.82
1.0
1.0
2.0
2.1
2.1
1.9
1.0
1.0
1.0
2.9
11.5
10.5
9.5
8.5
7.5
6.5
5.5
4.5
3.5
2.5
1.5
0.5
-0.5
BRUKER
NAME Yasooj UN
EXPNO 1154
PROCNO 1
Date_ 20180524
Time 18.30
INSTRUM spect
PROBHD 5 mm PABBO BB-
PULPROG zg30
TD 65536
SOLVENT DMSO
NS 20
DS 0
SWH 8012.820 Hz
FIDRES 0.122266 Hz
AQ 4.0894966 sec
RG 181
DW 62.400 usec
DE 6.50 usec
TE 293.9 K
D1 4.00000000 sec
TD0 1
======== CHANNEL f1 ========
NUC1 1H
P1 14.00 usec
PL1 -2.00 dB
PL1W 11.86359406 W
SFO1 400.2236020 MHz
SI 32768
SF 400.2200000 MHz
WDW EM
SSB 0
LB 0.30 Hz
GB 0
PC 1.00

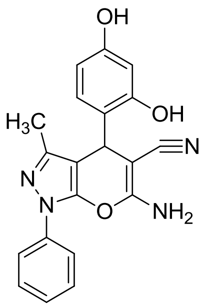
OH
OH
H3C
N
N
O
NH2
N

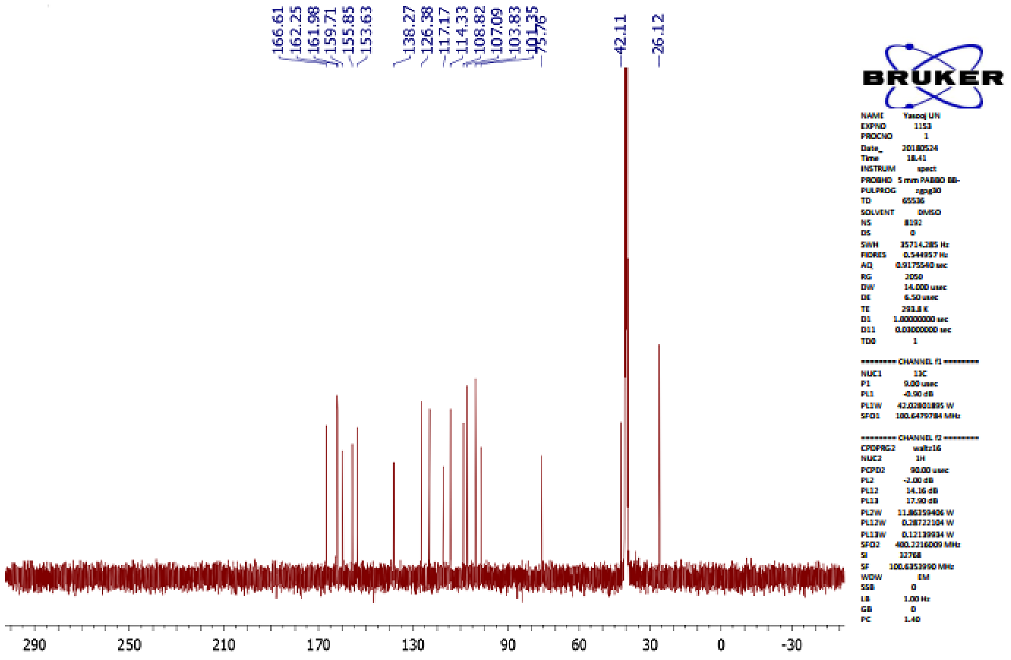
166.61
162.25
161.98
159.71
155.85
153.63
138.27
126.38
117.17
114.33
108.82
107.09
103.83
101.35
75.76
42.11
26.12
BRUKER
290
250
210
170
130
90
60
30
0
-30

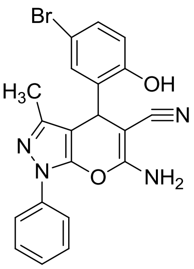
Br
OH
H3C
N
N
N
O
NH2

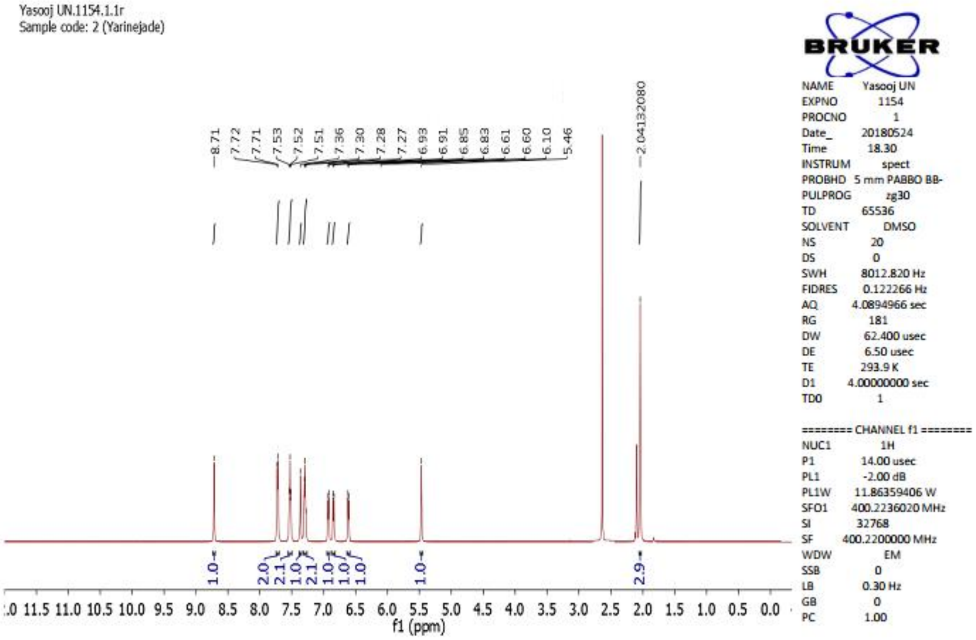
Yasooj UN.1154.1.1r
Sample code: 2 (Yarinejade)
BRUKER
NAME Yasooj UN
EXPNO 1154
PROCNO 1
Date_ 20180524
Time 18.30
INSTRUM spect
PROBHD 5 mm PABBO BB-
PULPROG zg30
TD 65536
SOLVENT DMSO
NS 20
DS 0
SWH 8012.820 Hz
FIDRES 0.122266 Hz
AQ 4.0894966 sec
RG 181
DW 62.400 usec
DE 6.50 usec
TE 293.9 K
D1 4.00000000 sec
TD0 1
======== CHANNEL f1 ========
NUC1 1H
P1 14.00 usec
PL1 -2.00 dB
PL1W 11.86359406 W
SFO1 400.2236020 MHz
SI 32768
SF 400.2200000 MHz
WDW EM
SSB 0
LB 0.30 Hz
GB 0
PC 1.00
8.71
7.72
7.71
7.53
7.52
7.51
7.36
7.30
7.28
7.27
6.93
6.91
6.85
6.83
6.61
6.60
6.10
5.46
2.04132080
1.0
2.0
2.1
1.0
2.1
1.0
1.0
1.0
1.0
2.9
f1 (ppm)

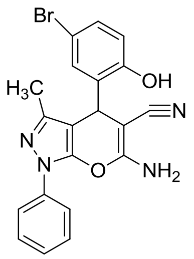

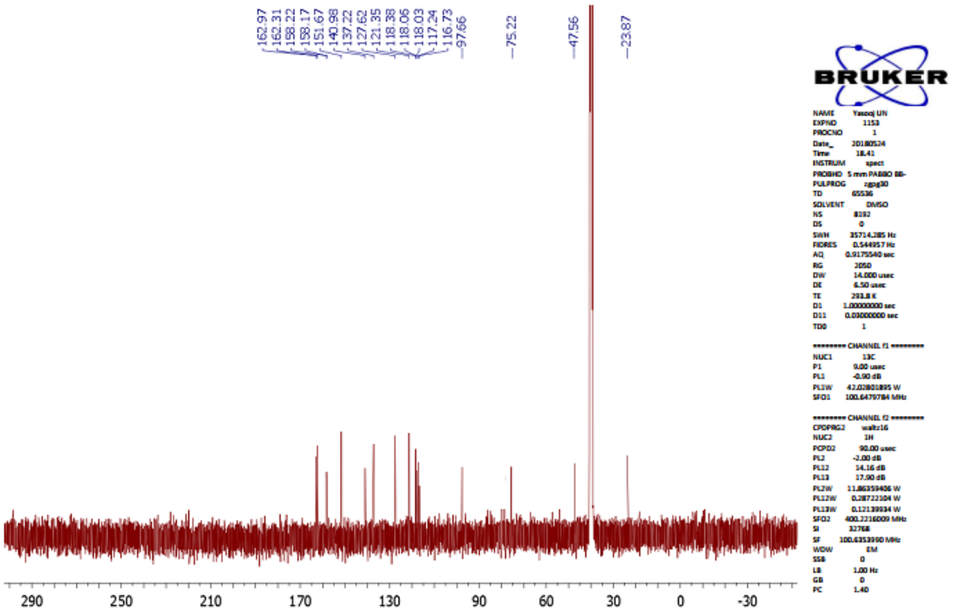

Supplement: RA-008-C8RA06886K-s001 [file RA-008-C8RA06886K-s001.pdf]
